# A model proposal for qualitative data analysis, interpretation, and reporting: contextuality, reflectivity, and narrativity

**DOI:** 10.1017/S1463423624000562

**Published:** 2024-10-28

**Authors:** Mehmet Ali Gülpınar

**Affiliations:** Faculty of Medicine, Eastern Mediterranean University, Famagusta, Turkey

**Keywords:** Qualitative paradigm, qualitative studies, research design

## Abstract

**Rationale:**

From education to healthcare and management processes, it is important to address the experience in health within its own complexity, context, and uniqueness. At this point, qualitative studies come to the fore and this increases the need for practical guides and models for qualitative studies. Qualitative studies have a paradigm that is different from quantitative research and its paradigm ontologically, epistemologically, and methodologically. These differences are reflected in the design of the research as well as the analysis, interpretation, and reporting of qualitative data. From such a point of view, this paper first briefly outlines the design process of qualitative studies and then proposes a model for the analysis, interpretation, and reporting of qualitative data.

**Conceptual/theoretical framework:**

The three core concepts of the model are ‘contextuality’, ‘reflectivity’, and ‘narrativity’. Such a conceptual/theoretical framework transforms qualitative data analysis, interpretation, and reporting processes into processes that are carried out with a reflective approach within their specific contexts.

**Model:**

Taking this into account, by considering a contextual, reflective, and narrative approach, two frameworks, namely, the ‘Contextual (Multiple) Reading and Analysis Framework’ consisting of three stages and seven steps, and the ‘Contextual Understanding, Interpretation and Reporting Framework’ consisting of four stages, were developed. This provides a practical guide to contextual and reflective data analysis, interpretation, and reporting for the use of those conducting qualitative studies.

From primary healthcare to education and governance processes, it is important to address all health-related experiences in their integrity, complexity, and context, with their behavioural, social, and humane dimensions. This challenging situation brings with it a new approach to education, healthcare, and health research: a holistic and contextual approach. At this point, qualitative studies come to the fore and this increases the need for practical guides and models for qualitative studies. In this regard, the present paper proposes a model for the analysis, interpretation, and reporting of qualitative data within the framework of contextuality, reflexivity, and narrativity.

## Qualitative studies

Quantitative and qualitative research, with their distinctive paradigms, core concepts, foci, and methods, address different aspects of the situation to be researched. Therefore, each has its own strengths and weaknesses. For this reason, it is important to take these aspects of research into consideration and use them appropriately (Cleland, 2015). Despite the emphasis on objectivity, objective reality, objectification, and quantification in quantitative research, which is based on a positivist and reductionist approach, the emphasis in qualitative research, which has a different paradigm, is on understanding and interpreting the experience (situation and phenomenon) studied in its integrity, complexity, authenticity, and contextuality. In quantitative research grounded on theories and hypotheses, there is a subject–object relationship. Associative relationships and causative relationships between variables are tried to be revealed. An effort is made to handle, explain, generalize, predict, and control the de-contextualized situation. In qualitative studies, which prioritize experience and proceed through research problems determined within the framework of existing theories, holistic phenomena/situations are handled in their own quality and singularity within intersubjective interaction and transformation (intersubjectivity). Efforts are made to describe, understand, and interpret; to explore and elucidate in depth. It is aimed to reveal intrinsic tendencies, affinities, attitudes, and behavioural patterns; and also, similarities and differences in understanding and making sense of phenomena. Furthermore, the quality of quantitative and qualitative studies is evaluated on the basis of different criteria. While the criteria used in quantitative research are ‘internal and external validity’, ‘reliability’, and ‘objectivity’, the evaluation of qualitative research is based on different ones such as ‘credibility’, ‘transferability’, ‘dependability’, ‘confirmability’, and ‘authenticity’ (Creswell, 2012; Creswell, 2013; Cleland, 2015; Mann & MacLeod, 2015; McMillan, 2015). Therefore, in the qualitative research process, it is important to consider the limitations of qualitative research as well as the differences between qualitative and quantitative research. The limitations of qualitative research include the following: not being sufficiently understood and accepted in the scientific community from time to time due to its different paradigm and process; difficulties in dealing with intensive qualitative data, labour-intensive, and time-consuming analysis process, requiring a meticulous process and management; weak explanatory and generalizing power; and more dependability of the analysis and interpretation processes on the individual skills of the researchers, therefore, it is not easy for the researchers to take the scientific distance in the process and is more prone to personal biases and idiosyncrasies (Anderson, 2010; Queirós et. al. 2017).

Therefore, it is critical that qualitative studies are designed, implemented, and reported taking into account these paradigm-level differences (different ontology, epistemology, and methodology). Qualitative studies should not be reduced to the use of qualitative data collection methods and tools; they should be differentiated from mixed design research in which quantitative and qualitative methods and tools are used together. Qualitative studies are research studies designed within a different paradigm with a different understanding of reality and knowledge, different research designs, research methods, and tools. It is not limited to the collection, analysis, and presentation of qualitative data obtained by using qualitative data collection methods and tools.

## Design of qualitative studies

Three points are critical in the design of qualitative studies: establishing a coherent macro and micro-framing, drawing the research model or conceptual/theoretical framework, and identifying the research problems. In this section, first of all, explanations on these three points are explained under the following three subheadings: ‘establishing a macro and micro framing’, ‘constructing the conceptual and theoretical framework’, and ‘determining the research problems’.

## Establishing a macro- and micro-framing

Establishing an integrated framing, from the approach chosen to the theories, from the research design selected to the data collection methods and tools will be decisive for the quality of the qualitative studies to be conducted (Bezuidenhout & van Schalkwyk, 2015; Bleakley & Cleland, 2015; Mann & MacLeod, 2015). A design without a macro-framework at the paradigm level, a well-defined theoretical framework, and appropriate research design with method and instrument choices will result in an incomplete and problematic qualitative study.

For the macro-framing of qualitative work, there are approaches with different ontologies and epistemologies (understandings of reality and knowledge) and foci, such as internal/cognitive constructivism, sociocultural constructivism, and structuralism; existential, experiential, phenomenological, and interpretivist/hermeneutic approaches; and narrativity, complexity, socio-material, Marxist, feminist, and critical approaches. The second framing is done by using the theories and models available in the literature concerning the situation or phenomenon that is the subject of the qualitative study. At this point, rather than general approaches, there are more specific theories and models such as Experiential, Transformational Learning Theories (Kolb, Mezirov), Activity Theories (Cultural-Historical Activity Theory), Actor–Network Theory, Flow Theory (Mihaly Csikszentmihalyi), Situated Learning, Emotional Singularity, Community of Practice, and Cognitive Load Theory. The theoretical framework of the study is established through a detailed literature review and reading. Research designs in the literature for qualitative studies include narrative study, action research, participatory action research, ethnographic study, phenomenology, grounded design, and case study. Qualitative data collection methods and tools include observation, participant observation, one-on-one in-depth interviews, focus group interviews, a special interview technique based on metaphors (Imaginative Metaphor Elicitation), reflective sessions, reflective writing, narrative writing, document analysis, text analysis, etc. (Creswell, 2013; Bleakley & Cleland, 2015; Fenwick & Nimmo, 2015; Mann & MacLeod, 2015; Nardon & Amrita, 2021). The design of the qualitative study is revealed by determining the most appropriate research design and methods among these.

In the design of qualitative studies described above, two components are critical: establishing the conceptual framework and identifying the research problems. The position of these two is at the heart of the design. It is at the centre of the implementation, analysis, and reporting processes of the qualitative study (Creswell, 2012; Bezuidenhout & van Schalkwyk, 2015).

## Constructing the conceptual and theoretical framework

When writing the qualitative study proposal, the theories identified through a rigorous literature review and reading are discussed in the introduction. The concepts, structures, components, and dimensions of the theories and the interactions and modelling between them are explained. Then, taking these theories into account, a model (research model) or framework (conceptual/theoretical framework) specific to the qualitative study is constructed. With this, the core concept set of the study is determined; the relationships between variables are visualized, the links between the main structure, components, and dimensions are revealed; the essence of the phenomenon/experience in the phenomenological study is visualized. In the design process of the qualitative study, the model, or conceptual/theoretical framework of the research, which is drawn as a draft, is finalized by improving it in line with the findings obtained during the analysis and interpretation of qualitative data. If a model or framework could not be drawn at the design stage, it emerges during the analysis and interpretation of qualitative data.

## Determining the research problems

**Figure 1.**
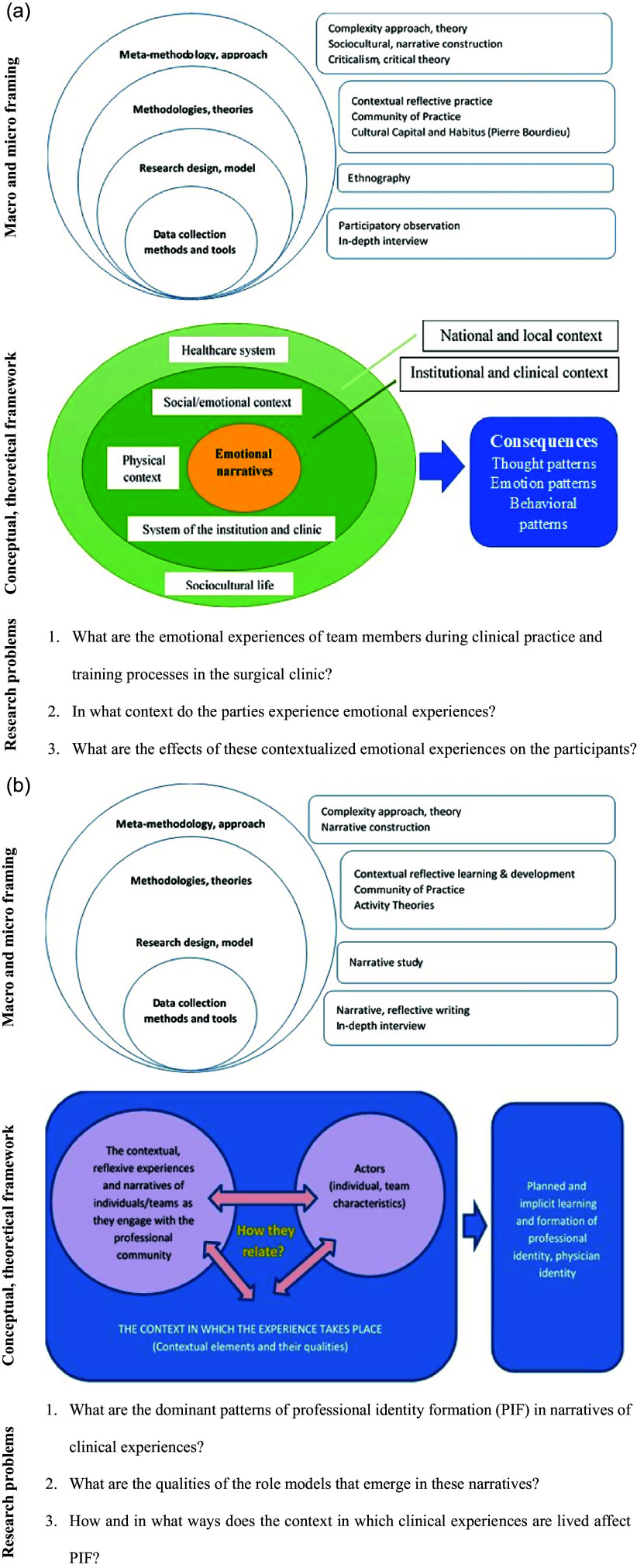
(a) Ethnography study on the healthcare and clinical education experiences of the surgical team. (b) A narrative study on professional identity formation.

After the conceptual/theoretical framework of the qualitative study is drawn, research problems are determined. The conceptual framework and research problems drawn specific to the research are at the heart of the methodology, data collection, analysis and interpretation, and reporting processes. They give direction to these processes and specify their foci. However, at the same time, in these processes, which should be carried out with a reflective approach, the conceptual framework and research problems are repeatedly reviewed and reorganized/improved with the experiences and outputs. What has been said so far is illustrated with an example in Figure 1a and 1b.

## Analysis, interpretation, and reporting of qualitative data

Data analysis and reporting in qualitative studies begin with data collection based on the conceptual framework and research problems determined. They continue with the analysis and interpretation of the data through multiple readings. It is important that this process is handled in a reflective continuum of deepening reading, focusing, meaning-making, and interpretation rather than a repetitive mechanical process (Srivastava & Hopwood, 2009). In this way, throughout the qualitative study, a reflective process is experienced in which the research problems, data collection and analysis, conceptual/theoretical framework, meaning-making, and interpretation processes are repeatedly reviewed, evaluated, and reorganized (Creswell, 2012; Creswell, 2013).

According to Srivastava and Hopwood (2009), the process of reflective analysis is driven by three main questions: (1) what does the data tell us, (2) what do I want to know (the research questions identified, the theoretical frameworks based on), and (3) the dialectical relationship between what the data tell us and what I want to know. In other words, it is possible to frame this process as a reflective process of interaction and transformation between experience (narratives, data) and theory (existing theories). Through such a process, the focus of the research, the research questions, and the conceptual framework are refined and further refined through reflection and reorganization of the qualitative study.

In the literature, there are various concrete proposals and models for the analysis and interpretation of qualitative data. Data analysis and presentation processes for different qualitative research approaches (narrative, phenomenology, grounded theory, ethnography, and case study) are described in detail, taking into account the characteristics of the approaches (Ajjawi Higgs, 2007; Creswell, 2012; Creswell, 2013; Cleland & Durning, 2015). In general, it is possible to summarize the process through the six steps defined by Creswell: preparing and organizing the data for analysis; initial exploration of the data through the process of coding it; developing a more general picture of the data (descriptions and constructing themes); representing the findings; interpreting and reporting the findings; and validating the accuracy of findings (member validation/checking, external audit, expert opinions/panel, and data and theoretical triangulation). As a more specific model, the stages in Ajjawi and Higgs’ six-stage model for hermeneutic analysis are as follows: immersion (pre-interpreting and getting a general sense of the data); understanding (deeper understanding and coding through the participant’s own statements; abstraction (identifying sub-themes); synthesis and theme development; and illumination and illustration of phenomena and integration and critique of findings within the research team and externally (confirming the appropriateness of the themes and reporting) (Ajjawi & Higgs, 2007; Bynum *et al.*, 2021).

## A qualitative analysis and interpretation proposal: contextual, reflective, and narrative model

While qualitative data analysis and interpretation processes are framed through reflectivity, the emphasis on contextuality and narrativity is not sufficient. However, it is important to analyse and interpret experiences through their unique contexts and narratives. In the model proposed in this paper, a contextual, narrative as well as reflective approach is adopted. Therefore, the analysis, interpretation, and reporting processes are framed as narrative, contextual, and reflective processes.

## Conceptual and theoretical basis of the model

The three core concepts of the model are ‘contextuality’, ‘reflectivity’, and ‘narrativity’. Based on these three concepts, a framework for the analysis, interpretation, and reporting of qualitative data was developed.

### Contextuality

It is important to consider all processes related to education, including qualitative studies, from educational change to curriculum development, learning, assessment, and evaluation processes, within the framework of the ‘complex social system’ approach with its own complexity and contextuality (Gülpınar, 2021; Gülpınar, 2024; Bleakley & Cleland, 2015). Complex social systems are process-focused and context-dependent systems, where process and context are intertwined. In this approach, where individuals and teams are accepted as actors with will, it is more appropriate to talk about co-framing and co-transforming, where the context and actors transform each other in contextual experience processes that progress through ‘mutual interaction and transformation’ rather than the linearity and determinism (non-linear and non-deterministic approach) of the natural, structural, sociocultural, or contextual (Gülpınar, 2021).

In general, contextuality is mirrored in this model in the following ways:

The first implication for qualitative data analysis is to transform the reading and analysis of qualitative data into a contextual one. In this direction, the following two points should be added to the analysis process: what are the ‘contextual characteristics’ in the text (i.e. what is the context of the experience/narrative) and how do the experiencing parties ‘relate to the context’ (see Table 2. Contextual text reading and analysis template).

**Table 2.** Example of contextual text reading and analysis

Narrative 1: Hesitant, scared comings, embarrassed, angry leavings			
*First reading: Impressions, questions*	*Experience/narrative* *Re-arranged text*	*In-depth reading and analysis*	*Reflections of the researcher, person analysing*
Work focused AND Blindness to the human situation, emotions, and culture Both for patients, their relatives, and for physicians and learners Inhumane environment (physical, social)	*I was in the outpatient clinic during the first week of my urology internship. Seven of my friends and I entered the outpatient clinic door at 9.30 in the morning. Our outpatient clinics were behind locked doors and designed to let in only the patients whose turn it was. As we entered, we showed our coats and cards to the attendant at the appointment desk. It was quite small and consisted of 4 rooms with no windows. Our specialist was already seeing patients in the room at the far end, which was bigger than the others. He had been seeing patients non-stop since 8 am and it was clear that he was already getting tired. When he saw us, he cheered up and invited us into the room with a smile. In that tiny room, eight of us sat on stools by the door and waited for the new patient to arrive. While the patient is inside, our specialist is only interested in him/her. He would later tell us the patient’s history and which tests he wanted and for what purpose.* *About an hour later, a young couple entered the polyclinic door. The couple looked around in the short corridor and our doctor called their names and called them into the room. The couple in their mid-20s entered the room with hesitant steps, holding hands. They stood and waited for guidance. The woman was waiting almost hidden behind her partner, never lifting her face from the floor. While they were standing, not knowing what to do, our expert was reading information about their previous arrival on the computer. Without looking up from the computer, he asked them to hand him the barcode. As the woman was rummaging through her purse, she dropped her wallet on the floor and her belongings scattered on the floor. After barely handing over the barcode, she hurriedly picked up her belongings. As the woman picked up her purse, our doctor looked up for the first time and smiled at the couple and told them they could sit down. A little more relaxed, the couple moved to the two chairs opposite. Neither of them had looked at us at all since they entered. After three minutes, which seemed like an infinite time to everyone in the room, our specialist asked them why they had visited.* *The man started talking saying that his name was M. and the lady next to him was A. and that they had been married for 3 years. His speech, which started quite confidently, started to shake as he got to his complaints. His voice got lower and lower and avoiding eye contact, he said that he had returned from the army 2 years ago, that he and his wife were trying to have a child, that they first went to a gynecologist and had her checked. He continued speaking in a shaky voice. Since all her wife’s tests were normal and suitable for conception, he had now consulted a urologist. When he finished, he looked up for the first time and said in a bold and clear tone that he wanted to have children and would do whatever needed to be done.* *Our specialist told him in a gentle tone that varicocele should be excluded first in male infertility and told him to go to the next room, take off his clothes and wait for us. 1–2 minutes after Mr. M. left the room, he stood up and put on his gloves. Turning to us, he said, ‘Come on friends, what are you waiting for? You have to see this’. We stood up eagerly at the same time as if we had agreed because none of us had ever seen a varicocele examination before. As the 9 of us headed to the other room, his wife Mrs. A. looked up at us in horror for the first time since she entered the room. She watched as we all took turns crowding into a stuffy room. He did not know what a varicocele examination was, but he understood the general concept. His cheeks turned red and his shoulders slumped as if in defeat. When we went into the other room, Mr. M. stared at the wall without looking at us. Like his wife, he prepared as if he wanted to say something, but he said nothing. Our specialist explained to us in detail and performed a varicocele examination. It was obvious from his body language that Mr. M. was very uncomfortable. He was fidgeting all the time and shaking his head from right to left as if to say how come. At that moment, it was obvious that Mr. M. wanted us not to be in that room. Neither he nor his wife wanted us in that intimate moment. Be sure that none of us wanted to be in that room after seeing their discomfort. Even one doctor was too much for them, and there were 9 of us, but our specialist didn’t seem to notice any of them. He didn’t even ask them if we could go in. After the examination, Mr. M. hurriedly got dressed. He never looked at his wife or us. Our specialist was sending them away after ordering some tests when Mr. M. gathered his courage and asked us not to be in the room when they returned. His tone was quite harsh and demanding. Our specialist immediately took his guard up and said in the same tone that this was a university hospital and if they did not want this, they would not come here. He continued to complain in a harsh tone that he was coming here and demanding something. During this whole process, we were sitting in our seats guiltily, both unhappy for disturbing the patient and for putting our specialist in this situation. At that moment, everything felt like our fault. The young couple walked out of the doctor’s office, now nervous and embarrassed.* *And at the end of the day, everyone got their wish. In the midst of his hundreds of tasks, our specialist had forgotten that the argument had even happened this morning. When the young couple returned, we weren’t really there. We were somehow invisible in this event in which our presence was the protagonist. We were just casualties in a war in which everyone was on guard and prepared.*	**Codes** Work orientation Intrusive computer and processes Constant patient care, fatigue Failure to see the situation, emotions, and culture of the patient and his/her relatives: privacy, shyness, shame, inability to have children in the culture, patriarchy? An angular, sharp language, and discourse over power	During this experience, there was a huge crowd in the corridor of the polyclinic and in front of the door. The doors were locked because someone kept poking their head in. During the analysis, I thought about how I relate to cultural and emotional situations during my own experiences in healthcare processes, how much I should or should not include them in the process, and my ambivalence at this point.
		**Metaphors** A war where everyone is on their guard Three minutes that seemed like forever Sitting guiltily in your seat Invisibility	
		**Potential themes** Clinical workload, work-orientedness (routine, technical processes), and the de-humanization Emotional, culturally unsafe encounters Authoritarian language and discourse in the patient–physician relationship	
		**Contextual elements, features** *Physical, social, relational context:* Locked doors, sequential, authorized entrances Four small windowless rooms, 9 people where even 1 person is too many Relating through work, not seeing what is humane, what is cultural *Social, cultural context:* Power relationship/authoritarian relationship, authoritarian, reactive discourse, and behaviours, e.g. Not seeking consent ‘This is a university hospital…’ ‘He both comes here and wants something’	
		**How it relates to the context** Instead of expressing and sharing feelings, it is a cumulative, explosive, charged reactive way of relating: Tolerating boredom, embarrassment, and finally responding explosively (patient and physician) Patient: ‘don’t let them be in the room when we get back…’ Physician: ‘This is a university hospital…’ ‘he both comes here and wants something’ Taking your guard up in battle Relating by being silence, storing up (students): Silence, guilty standing, and invisibility	

Another implication is the suggestion of ‘multi-layered reading and analysis’. In multiple readings, in the first analysis, two to three main context domains are identified based on the above two considerations (contextual characteristics, the way of relating to the context). Then, by returning to the text, the data can be subjected to multi-layered reading and analysis in the focus of each determined context separately. This type of reading allows for more focused and in-depth data analysis.

The contextualization of interpretation and meaning-making processes is another implication in this way. By taking the context into account, this process is addressed at three main points: (1) understanding the researched situation in its own context (contextual understanding), (2) interpreting the researched situation in different contexts in the light of the findings in the literature and the contexts of these findings (multiple understanding, interpretation, and meaning-making in different contexts), and (3) proposing a new context, addressing the situation through it, and developing new frameworks and suggestions for its solution (re-contextualization and re-framing).

The last implication in this direction is the inclusion of the heading ‘Contextuality and Reflexivity’ in the methodology section of the report. Under this heading, the context in which the qualitative study takes place (institutional, local, social, national, etc.) and the context of the research and the research team itself are clarified. In this way, the qualitative study is strengthened in terms of many criteria (confirmability and transferability).

### Reflectivity

**Figure 2.**
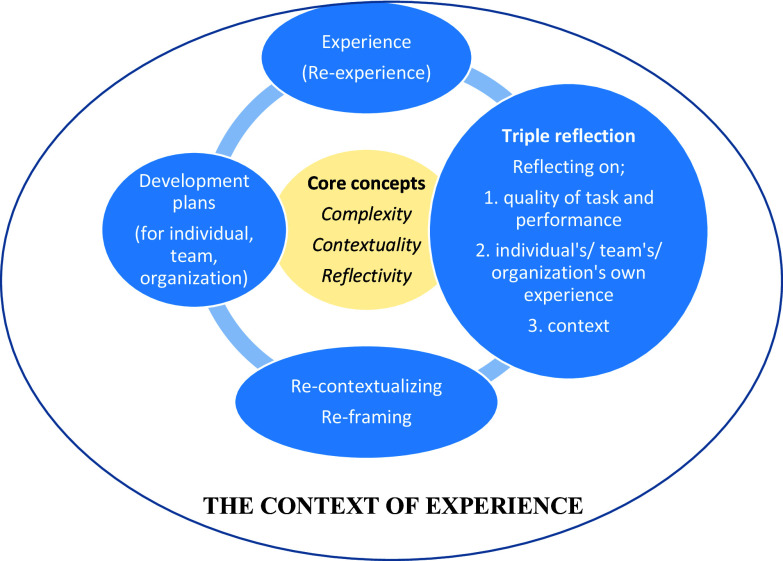
Contextual reflective practice with triple reflection.

Contextual reflective practice is a practice in which the parties look at their experiences and the context in which the experience takes place from a certain critical distance and reflect on these. In this way, they experience a practice in which they develop and transform by learning from concrete experiences, and through this, they transform both the practice and the context in which it takes place. In reflectivity, it is important to start from a concrete experience and return to it. It is a reflection on experience and the context in which the experience takes place rather than a reflection on thought as in philosophy. Another important point is the encounter between experience (concrete) and existing theories (abstract) and the transformation of both theory and experience together as a result of these encounters. As can be seen in Figure 2, contextual reflective practices are continuous contextual and reflective processes of experiencing, reflecting on experiences and their contexts, re-contextualizing, re-framing, and re-experiencing. So, it is important to conduct qualitative studies as reflective processes. In such a reflective process, the qualitative study becomes stronger in terms of criteria such as credibility, confirmability, and transferability.

### Narrativity

Individuals, teams, and organizations construct their individual, professional, and organizational meaning-makings, realities, professional identities, and organizational cultures through narratives of their contextual and reflective experiences (narrative construction of reality, self, and professional/organizational identity). Therefore, narrativity has recently come to the forefront as a different approach in all fields from healthcare and education to change and leadership processes (Bruner, 1991; Bruner, 2006; Charon, 2007).

A similar situation can be mentioned for qualitative studies. As will be remembered, qualitative studies conducted on the institutional and social experiences of an individual, a team, or a particular community focus on the uniqueness and subjectivity of experiences. It puts the effort to understand and interpret them at the centre. Therefore, understanding and interpreting the situations, processes, and outcomes of these experiences requires being open to the narratives of individuals, teams, institutions, and communities. It emphasizes prioritizing narratives over existing theories in the processes of understanding. It also emphasizes staying in the experience and narrative and returning to the narrative again and again, while also seeing existing theories as an important component of the process of understanding. The most important trap at this point is to make a superficial reading and analysis by foregrounding existing theories, and in this way, reproducing what is already present in theory.

Narrativity and reflectivity can be translated into qualitative study processes in several different ways. Reflective, narrative writings, and reflective researcher diaries written by the researcher are some of them. Through reflective/narrative writings and the researcher’s diary, the researcher writes their own narratives about the implementation and data analysis processes and their reflections on their experiences. They take notes on the process and explain the changes made during the process with their justifications. Another way is to write narratives during the reporting process. With this, the qualitative study will become stronger in terms of multiple criteria (credibility, confirmability, and authenticity).

Narratives can be written in the following three sections in reporting:

‘Contextuality and reflexivity’ in the Methods section, where the primary researcher narrates how they experienced the qualitative research process with its contextual dimensions.

In the ‘Findings’ section, re-narrating one to two representative experiences in the words of participants in a way that vividly reflects the experience and the context in which the experience was lived.

In the ‘Interpretation and suggestions’ section, the narration of a sample experience (re-experiencing) through the re-contextualization and re-framing that emerged as a result of the interpretation.

## Contextual, reflective, and narrative model

In this model, developed through a triple approach, the ‘qualitative data analysis and interpretation process’ and the ‘reporting process’ are addressed separately and two concrete frameworks are developed.

### First framework: a three-phased contextual (multiple) reading and analysis framework

The first framework based on contextual reading and analysis of qualitative data, the ‘Three-phased Contextual (Multiple) Reading and Analysis Framework’, consists of two different types of analysis. These are ‘single-layered’ and ‘multi-layered’ reading and analysis. In multi-layered reading and analysis, which is presented as a different reading method, there is multiple reading and analysis of the text by focusing on two to three different contexts. The process is carried out by reading, analysing, and interpreting the narratives related to the experience that is the subject of the research separately through two to three selected contexts in a layered and therefore more nuanced and in-depth manner. For example, in ethnographic research with a narrative design in which the narratives of the surgical team about their experiences in clinical education and healthcare processes are studied, the qualitative data collected can be subjected to triple reading and analysis through three different contexts, namely (1) social, emotional, relational context, (2) system, and (3) dominant discourse, language, and patterns of relating to power in institutional and social culture. A fourth layer can be in the form of reading and analysis on embodied predispositions, orientations, attitudes, and behaviours (habitus) and frequently used ‘metaphors’ in such a habitus.

The framework model, which consists of three phases and seven steps, is described below. The analysis process is carried out through the template prepared and an example of such a template is given in Table 1. An example of an analysis using this template is also presented in Table 2.

**Table 1.** Contextual text reading and analysis template

Narrative 1: …			
*First reading: Impressions, questions*	*Experience/narrative* *Re-arranged text*	*In-depth reading and analysis*	*Reflections of the researcher, the person analysing*
		Codes	
		Metaphors	
		Potential themes	
		Contextual elements, features	
		How it relates to the context	

#### Phase I: initial readings and analysis

##### Step 1 – pre-reading and analysis

In this step, the text is generally read from beginning to end and first impressions, thoughts, feelings, and questions are noted. It is critical that the analysis is done independently by at least two researchers (credibility). After reading the text, the analysers can ‘take a step back’ and ask themselves the following question: ‘What is this text/narrative mainly talking about, what does it mean?’ Other questions they can then ask themselves are: ‘Do we need more data to understand exactly what the experience (situation, phenomenon) is? Is it necessary to define an additional problem or sub-problem that is not defined in the research problem but emerges from the first readings?’

##### Step 2 – review the research problems, data collection process, and conceptual/theoretical framework

This step is done together with the research team. If the first reading points out issues for improving the research problems or the data collection process, required adjustments are made. The identified research problems are reviewed and re-defined. Plans are made to collect additional data. If data are collected through, for example, in-depth interviews, participant observation and/or narrative writing, additional interviews, writing, or observation are planned and conducted. When necessary, a new method or tool is included in the design to collect supporting data or to triangulate the data.

The conceptual and theoretical framework created/selected in the process of designing the qualitative study is reviewed, the literature is revisited, and if necessary, adjustments are made within this framework.

##### Step 3 – extended contextual reading and analysis

In this step, again independently by two researchers, the prominent experiences/narratives in the text are marked (fragmented, underlined, coloured, classified, and labelled), along with their main points/emphases and the contextual features in which the experience takes place, in line with the (reorganized) research problems. Codes and themes are then created based on these.

- *Creation of initial codes*: First, the text is segmented, labelled, and coded through repeated readings. Then, the codes that emerged first-hand are reorganized by combining, rephrasing, and eliminating some of them (25–30 codes). It is important to do this process by taking into account the research problems. Otherwise, the focus of the research will be lost through over-coding and this may result in the researchers getting lost in the text and codes.
- *Identifying the contexts in which the experience takes place, with their contextual features*: A similar reading and analysis is carried out regarding the contexts in which the experience/narrative takes place and the features of these contexts. In this regard, identifications are made and codes are created.
- Creating subthemes and themes: Based on the reorganized codes, these are transformed into more inclusive, abstract expressions to create subthemes and themes related to the experience and the context in which it was lived (five to eight themes of two to four words each). At this point, we returned to the theories determined during the design of the research, on which the research topic was based. The concepts of these theories can be utilized during the identification of themes.

However, there are two critical issues to be considered at this point. The first is to prioritize the experience and stay in the experience, and thus, instead of making visible a difference that emerges through analysis, to prioritize the theory, to quickly match the themes with the existing explanations and conceptualizations of the theories, and thus to miss the nuances and uniqueness that can emerge through qualitative work. Another common trap is to carry out a superficial reading and analysis and conduct theming as a way of collecting content and giving it descriptive titles. This is not what theming is about.

1. *Consensus on themes, determining main contexts, and dimensions*: Researchers who independently determine codes and themes come together at this point and reach a consensus on the outputs. The agreed themes are grouped among themselves to form the main contexts and dimensions. A third researcher may be engaged to ensure consensus at this point, and expert opinion may be consulted to refine the decisions. Again, in this step, it is important to select one to two people from different participant segments to confirm the relevance of the outputs and how well they match with the participants’ own experiences.

#### Phase II: framing/modelling

In this phase, the research team works together to construct the conceptual/theoretical framework or research model together with its contextual dimensions. It is critical to consult expert opinion at this point. Framing can be done in two ways. First, if there is no concrete conceptual, theoretical framework selected from the theories in the literature or created by the research team for the study, the framework is constructed here for the first time. The other is to reorganize the existing framework based on the analysis outputs. According to the results of the analysis, if it is seen that the existing framework is working, the necessary modifications are made.

##### Step 4 – organizing/creating the conceptual, theoretical framework

Taking into account the two to three main contexts (which frame the experience in various aspects, differentiate, transform and give meaning to the experience with its process, outcome and effects) and themes determined by consensus in the previous step, the conceptual/theoretical framework of the research (or the model of the research), which was initially created in the design process, is reorganized. If the framework/model was not created during the design process, it is presented for the first time in this step. At the same time, in this step, if multiple/layered reading and analysis will be carried out over two to three contexts in the next advanced analysis phase, these layers will also be visualized.

##### Step 5 – revision of the research problems

Based on the emerging or revised conceptual/theoretical framework and the two to three main contexts of the experience, the research problems are reviewed and revised if necessary.

#### Phase III: advanced reading, analysis, and framing

##### Step 6 – advanced reading and analysis

In this step, further reading and analysis is carried out independently by two researchers. One of the following two methods is used in this reading: (1) single-layered contextual reading and analysis, and (2) multi-layered reading and analysis through two to three main contexts identified. If the content in the narratives of the two to three contexts is dense and sufficient, layered reading may be preferred. In this way, with two to three separate readings, each specific to a main context, more focused, in-depth, and fine-grained analysis of the text will be possible through these layers; themes and sub-themes will be determined context-specifically.

- *Single-layered reading and analysis*: The text is returned to and read again through the emerging framework (with its contextual dimensions) and the revised research problem. With this reading, the themes, dimensions and contexts agreed upon in the previous stage are checked, supporting and contradicting quotations/data are marked, revised if necessary, and new themes are identified, if any. In this way, both the relevance of the analysis outputs is tested (verification, confirmation) and the outputs are finalized. In this process, sample quotations to be used in the report are also selected.
- *Multi-layered reading and analysis*: Based on the two to three main contexts identified in the previous stage, the text is read and analysed separately for each context. Themes and sub-themes are constructed specific to the context in question. Again, if necessary, the research team can come together to create a separate conceptual framework for each main context.

##### Step 7 – finalizing the conceptual/theoretical framework of the study

After single or multi-layered advanced reading and analysis, taking into account the outputs refined in step six, the research team comes together and finalizes the conceptual/theoretical framework of the study. The research problems are reviewed for the last time and adjustments, if any, are made. In this step, the conceptual/theoretical framework can also be enhanced by seeking expert opinion or organizing an expert panel with a group selected from different fields of expertise.

Throughout the seven-step process, interpretation processes begin and evolve along with contextual and reflective analysis. However, by the end of the seventh step, it is time to intensify the process of contextual interpretation and reporting on the final outputs/findings. Within the scope of this model, a second framework has been developed in this sense and its details are presented below.

### Second framework: a four-phased contextual understanding, interpretation, and reporting framework

In this model, which was developed by assuming a different paradigm, the processes of understanding and interpretation are framed. Before moving on to the second concrete framework of the model, we first tried to draw a conceptual framework for contextual understanding. With this framework, which can be named as ‘Triple Contextual Understanding and Interpretation’, it is possible to differentiate the understanding and interpretation of qualitative studies as follows:

1. *Understanding in its context*: Here, the effort to understand the experience (situation, phenomenon) that is the subject of the qualitative study in its own context is dominant. This effort to understand starts with the process of analysing qualitative data and continues with the writing of findings in reporting. At this point, experience is prioritized rather than theory, and staying in the experience is predominantly considered during the process.
2. *Understanding and interpreting in different contexts*: At this point, theory as well as experience is taken into account, and the experience that is the subject of the qualitative study, the findings of the qualitative study, and different approaches, theoretical frameworks, scientific studies, and their contexts in the literature are discussed and interpreted together. Here, there is an effort to understand, interpret and make sense together through different contexts, approaches, and theoretical foundations. The discussion/comment section in the report is written through such an effort of understanding and interpretation.
3. *Re-contextualization and re-framing*: The effort to understand and interpret in different contexts is completed at the third step with re-contextualization and re-framing (abstraction). In the process of analysing qualitative data, the re-framing that starts with the second stage (establishing the conceptual and theoretical framework) continues while writing the discussion/interpretation section in the report and is completed with the limitations, conclusion, and recommendations written at the end of the section. The reframing/contextualization of the qualitative study is evaluated through reflections on the contexts of the experience, the research design and the team; its strengths and limitations are discussed. Conclusions and recommendations are written through reframing.

**Figure 3.**
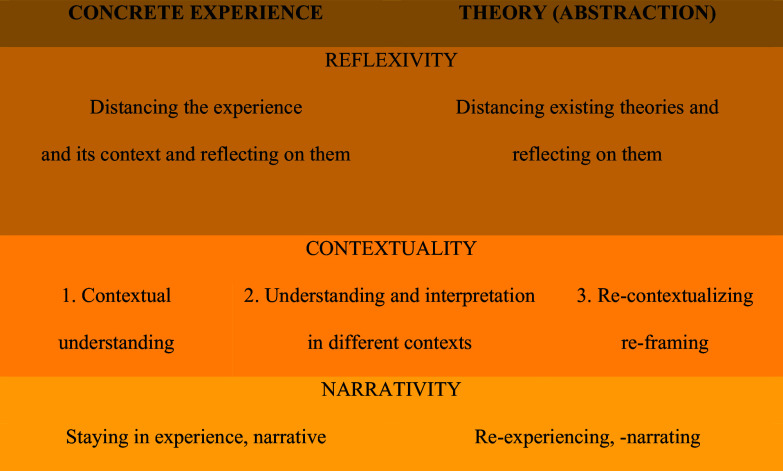
Contextual understanding and interpretation spectrum.

When concrete experience and existing theories from the literature (abstraction) are placed at the two ends of the spectrum, the position in this triple understanding and interpretation will shift from right to left as we move from the first to the third (Figure 3).

Considering this triple understanding and interpretation, a ‘Four-phased Contextual Understanding, Interpretation and Reporting Framework’ has been developed for the reporting process. These stages can be briefly summarized as follows:

#### Phase 1 – presenting the context of the qualitative study

The context of the qualitative study is presented in the methodology section of the report. This context can be presented in two ways.

- *Contextuality and reflexivity*: It is one of the indispensable headings of the method section in qualitative studies. Two types of explanations are given under this heading. The first is a detailed explanation of the context (institutional, social, regional, and national) of the experience addressed in the qualitative study. The second is a description of the team conducting the research. In the research, thick descriptions and reflections are written about who and what was done, the context in which the research was conducted, and the individual/team contexts of the researchers (Rudd & D’Andrea, 2015).

These contexts and reflections are also taken into account when writing the discussion and interpretation sections. All these contextual and reflective accounts strengthen the qualitative study in terms of multiple criteria (credibility, confirmability, and transferability).

1. *Narrative*: The primary researcher can write a short story/narrative about their experience, taking into account the diary they kept during the research process and their narrative and reflective writings, reflecting the context of the field, institution and society in which the experience took place. In this way, the qualitative research process is presented as alive with its context.

#### Phase 2 – understanding and presenting in context

When writing the findings section, the themes and subthemes that emerge as a result of the analysis are presented with sample phrases. Here, both the experience that is the subject of the research and its context are presented in detail. An effort is made to understand what kind of experience is lived in what kind of context, how the parties relate to this context, how and in what way this context and the way they relate affect the experience, and what the consequences are. Another way of presentation is to visualize the components, dimensions, patterns, weights, or interrelationships that emerge from the analysis (conceptual framework, model of the research, concept clouds, diagrams, etc.).

As in the first phase, narratives/stories can also be used here. However, in this case, the narrative/story is written with a different purpose. The aim is to re-narrate in the voice of one to two representative participants, using sample statements, one to two typical experiences that emerged from the analyses, in a way that vividly reflects the experience and the context in which the experience took place.

#### Phase 3 – understanding and interpreting in different contexts and presenting the situation subject to the research in multiple ways

While writing the discussion and interpretation section of the qualitative research, the findings of the study are compared with the literature and the situation subject to the research is tried to be understood and interpreted. Here, both the findings of the research and the context revealed by the research and other studies and theories in the literature are taken into consideration together and the situation in question is interpreted through different contexts, findings, and theoretical frameworks. Various aspects are presented in detail. Different approaches, theoretical frameworks, and the conceptual/theoretical framework that emerged in this study are critically evaluated with their possibilities and limitations. Furthermore, through reflections on the context of the research in question, the understanding, interpretation, sense-making, and inferences put forward in the study are evaluated together with their strengths and limitations.

##### Stage 4 – recontextualization, framing, and presenting recommendations based on this

With the theoretical/conceptual framework that emerges from the qualitative study, the experience (situation and phenomenon) that is the subject of the research is re-contextualized and theoretically reframed. In this way, it contributes to the literature to improve and transform both the experience that is the subject of the research and the existing theoretical frameworks related to it. Furthermore, in line with the new framework, recommendations are listed in the final section of the report.

Finally, a narrative can also be written at this stage. The purpose of narrative writing here is to help the reader visualize the sample 1–2 experiences (re-experiencing) through re-contextualization and reframing.

### Limitations and or challenge of proposed model

With its different paradigm, design, and implementation process, conducting qualitative research has various challenges and limitations. These challenges and limitations are discussed above. Within the scope of this model developed through the narrativity, contextuality, and reflectivity approach, this article has tried to concretize these approaches, which have been on the agenda recently, in the context of qualitative data analysis, interpretation, and reporting and to create a framework in this direction. Since the approaches are new and offer a different reading, analysis, and interpretation framework, and therefore the general reader may not be very familiar with them, there may be extra challenges in implantation. However, considering the importance of the approaches and the fact that this is the first article in this direction, it is thought that these challenges will be overcome in time and that the proposed framework/model will become more useful for the general user with new research and articles in this direction.

## Concluding

Practical guides on the analysis, interpretation, and reporting of qualitative data are useful tools for researchers who will work in this area. In this paper, a new framework is proposed based on contextualization, reflectivity, and narrativity and presented in a guide format for the evaluation and use of those interested. The proposal will be improved over time with the feedback of educational experts and users and will become more useful, thus contributing to the literature in this way.
